# Virtual touch tissue imaging and quantification: value in malignancy prediction for complex cystic and solid breast lesions

**DOI:** 10.1038/s41598-017-07865-7

**Published:** 2017-08-10

**Authors:** Ying Zhang, Chong-Ke Zhao, Xiao-Long Li, Ya-Ping He, Wei-Wei Ren, Cai-Ping Zou, Yue-Wu Du, Hui-Xiong Xu

**Affiliations:** 1 Department of Medical Ultrasound, Shanghai Tenth People’s Hospital, Ultrasound Research and Education Institute, Tongji University School of Medicine, Shanghai, 200072 China; 2 Department of Medical Ultrasound, Ningbo Medical Treatment Center Lihuili Hospital, Ningbo, Zhejiang Province 315040 China

## Abstract

This study was aimed to evaluatethe usefulness of conventional ultrasound (US) and US elastography, including the latest virtual touch tissue imaging and quantification (VTIQ), in malignancy prediction for complex cystic and solid breast lesions. Eighty-nine complex cystic and solid breast lesions were subject to conventional US and US elastography, including strain elastography (SE), virtual touch tissue imaging (VTI) and VTIQ. Among the 89 lesions, thirty-four (38.2%) lesions were malignant and 55 (61.8%) lesions were benign. Sixteen variables were subject to multivariate logistic regression analysis. Pattern 4b in VTI (odds ratio, OR:15.278), not circumscribed margin of lesion (OR:12.346), SWS mean >4.6 m/s in VTIQ (OR:11.896), and age elder than 50 years (OR:6.303) were identified to be independent predictors for malignancy. In receiver operating characteristic (ROC) curve analyses, associated areas under the ROC curve (Az) for conventional US could be significantly elevated, from 0.649 to 0.918, by combining with US elastography (*p* < 0.0001). The combined diagnostic method was able to improve the specificity (32.7% vs. 87.3%, *p* < 0.0001) without sacrificing the sensitivity (97.1% vs. 85.3%, *p* = 0.075). Both conventional US and US elastography contribute substantially to malignancy prediction in complex cystic and solid lesions. The diagnostic efficacy of conventional US in terms of Az and specificity could be significantly improved by combining with US elastography.

## Introduction

The incidence and mortality of breast cancer is high. American College of Radiology (ACR) Breast Imaging Report and Data System (BI-RADS) was originally established for breast imaging to standardize and unify terminology among different diagnostic approaches as well as different subjects. BI-RADS has been prevailing all around the world for its efficient risk stratification system and powerful diagnostic efficacy such as high sensitivity to realize early detection for breast cancer. Since being complemented into BI-RADS in 2003, conventional ultrasound (US), consisting of grey scale image and color Doppler flow image (CDFI), has been widely noticed for the cost-effective, non-invasive, non-radiation procedure and sensitive malignancy prediction^1–3^. In comparison with mammography, US is absolutely necessary when dealing with the complex cystic and solid breast lesions since its additional ability of identifying cystic component. The pattern of breast lesions named “complex cystic and solid” is a coexistence of cystic/fluid and solid component on US according to ACR BI-RADS US lexicon^4^, with a documented malignancy rate of 10–62%^5–10^. Besides, thick walls and thick septations (≥0.5 mm) are also involved^7, 8, 10^.

As to the diagnostic efficacy, conventional US is able to achieve high sensitivity (71.2–100%) in malignancy prediction with the specificity sacrificed somewhile (32–92%)^1, 11^. US elastography, the general appellation for a series of techniques that assessing tissue stiffness through US based methods, is becoming one of the most optimal candidates to improve specificity of conventional US. Numeral studies have observed that conventional US and adjunctive US elastography are able to improve specificity in malignancy prediction for solid breast lesions without sacrificing sensitivity and to decrease the possibility of unnecessary biopsy or operation^12–18^.

For several decades, US elastography has made remarkable progress in clinical practice, from strain elastography (SE) to shear wave speed (SWS) imaging. Recently, as the evolution of acoustic radiation force impulse (ARFI) imaging (i.e. Virtual touch tissue imaging, VTI; Siemens Medical Solutions, Mountain View, CA, USA) and point shear wave speed (SWS) measurement (i.e. Virtual touch tissue quantification, VTQ; Siemens Medical Solutions, Mountain View, CA, USA), a kind of SWS imaging (i.e. Virtual touch tissue imaging and quantification, VTIQ; Siemens Medical Solutions, Mountain View, CA, USA) has drawn attention because of the improvements made in terms of multiple-point measurement, shear wave quality map and smaller region of interests (ROIs) (i.e. around 2 × 2 mm). According to the diagnostic performance in solid breast lesions, it is reasonable to expect the contribution conventional US and US elastography, especially the latest VTIQ technique, made to malignancy prediction in complex cystic and solid breast lesions. However, limited attention has been paid to the application of US elastography which contains a potential ability of identifying cystic component. To our best knowledge, no study of evaluating complex cystic and solid breast lesions by VTIQ has been documented. Consequently, the present study was aimed to characterize malignant complex cystic and solid lesions with VTIQ technique.

## Materials and Methods

### Patients

With relevant guidelines and regulations complied, the current study was retrospectively approved by the Ethical Committee of the Shanghai Tenth People’s Hospital of Tongji University School of Medicine and informed consents were waived from all patients. From June 2014 to December 2015, one hundred and one complex cystic and solid breast lesions in 101 consecutive patients were pathologically confirmed through US-guided core needle biopsy (CNB) or surgery. Finally, eight-nine lesions in 89 patients (mean age, 45.4 +/- 15.8 yrs, range, 20–92 yrs) were enrolled by conforming to the following eligibility criteria: (1) Lesions should be fit for the definition of complex cystic and solid echo pattern in ACR BI-RADS US lexicon. (2) No treatment history. (3) Female patients. (4) Mammary gland originated. (5) Being the most suspicious one if multiple lesions occurred in one patient. (6) Images were complete, and clinical information was available in the institute medical record system.

### Conventional US and US Elastography

In the current study, the same Siemens S3000 equipment (Siemens Medical Solutions, Mountain View, CA, USA) was applied for all patients. The 18L6 linear array transducer (frequency range, 7–17 MHz) and/or 9L4 linear array transducer (frequency range, 4–9 MHz) were assigned to conventional US examination, and the 9L4 linear array transducer was assigned to VTIQ examination. Conventional US examinations of bilateral breast were performed by one of three board-certified radiologists. With optimized machine setting and sufficient gel applied, high quality images were acquired in patients’ supine position, including grey scale images and color Doppler images. US images on both transverse and longitudinal cross sections of each target lesion were obtained and stored in the internal hard disk for subsequent analyses. The longitudinal cross section is of the largest area in the lesion. And the transverse cross section is orthogonal to the longitudinal cross section. Maximal diameters of lesions were all measured on longitudinal cross section view.

Afterwards, the lesions were subjected to a series of US elastography techniques, including SE, VTI and VTIQ. During all the US elastography process, location of probe should not move. SE was firstly executed. With patient’s breath held for several seconds, the radiologist perpendicularly applied the probe on the surface of breast. The sampling box of SE was delivered to cover the target lesion and sufficient surrounding tissue once an optimal grey-scale view achieved. Meanwhile, a slight manual compression through the transducer was initiated by the radiologist with a frequency of twice per second. After scanning approximately 10 seconds, image was frozen. By reviewing several frames, a high quality SE image indicated by quality factor (QF) value above than 60 was selected to be stored and analyzed. Tissues in sampling box were coded by colors according to stiffness in SE mode, ranging from red (soft) to blue (hard) by default. In the current study, VTI and VTIQ were on the basis of ARFI technique. In ARFI mode, slight deformations of tissue in sampling box were excited by a short-duration acoustic pulse with fixed transmission frequency. The quantified information of tissue deformations could be directly denoted by the velocities of longitudinal wave (i.e. reflected echo) and transverse wave (i.e. shear wave) mechanically. The reflected echoes were ranked in grey-scale map in VTI mode according to the wave velocity, ranging from bright (soft/easy to make deformation) to dark (hard/hard to make deformation). And discretionary information of shear wave propagation speed was computed by SWS measurement (m/s) in VTIQ mode. For the VTIQ, firstly, the SW-quality image denoted by a two-dimensional color map was obtained to assess the quality of SWS imaging, ranging from green (high quality) to red (low quality). Thereafter, the color-coded VTIQ image was achieved, ranging from blue (low speed) to red (high speed) by default (0.5–10 m/s). The SWS values of solid component in the lesion were quantitatively interrogated by a fixed ROI. To increase the reliability and reproducibility of measurement, each lesion was measured seven times. There were several points should be noticed in delivering ROI. (1) The area coded by highest and lowest SWS should be selected, and remaining measurement was randomly selected. (2) Areas with liquid, macrocalcified or adjacent tissue were not allowed to be selected. (3) The area indicated to be low quality according to SW-quality map should be avoided. Readjustment of the scale was needed in case of the occurrence of “higher” or “lower” values. Usually, radiologist may spend about 5–10 minutes on the whole procedure without any inconvenience or additional cost for each patient.

### Image Interpretation

Images were retrospectively reviewed by two skillful radiologists with consensus, who had similar experience in conventional US and US elastography. All the radiologists were blind to the pathological results. For internal content, lesions were identified to be predominantly solid (<50% cystic) and predominantly cystic (≥50% cystic) on conventional US. Terms and subcategories in ACR BI-RADS US lexicon, including shape (oval, round, irregular), orientation (parallel, not parallel), margin (circumscribed, not circumscribed), echo pattern (for solid component) (hyper-, iso-, hypo-, heterogeneous), posterior acoustic features (shadowing, enhancement, combined pattern, no posterior acoustic features), internal vascularity (absent, present), internal calcification (absent, present), were referred and assigned to each lesion. Meanwhile, risk stratification of each lesion was assessed by radiologist in ACR BI-RADS category. Concerning the following clinical options (i.e. follow up or biopsy /excision), the lesions assigned to be BI-RADS 1–3 category were regarded as probably benign which were dealt with follow up, while 4a-5 category were probably malignant which were recommended to biopsy/excision. With respect to SE, Tsukuba score was used to assess the stiffness of solid component in a five-points scale^14^. The higher score might accompany with the stiffer tissue. In VTI mode, a method advocated by Tozaki, *et al*. was applied in ranking lesions into 5 level patterns^9^: Pattern 1: Neither bright nor dark area is found inside the lesion (i.e. the entire target region appears grey). Pattern 2: A bright area appears on the target region. Pattern 3: The lesion shows a mixed pattern of bright and dark area, including dark septations and dark rim. Pattern 4a: Darkness occupies most of the lesion, but not the whole. Pattern 4b: Darkness occupies the entire lesion, and surrounding tissue is involved. In VTIQ mode, the max, mean, median and min of SWS values were obtained and recorded for analysis respectively.

### Consistency on conventional US and US elasography among Intra- and Inter-observer

To evaluate inter-observer reproducibility on conventional US, SE and VTI, images of 30 consecutive patients which have not been enrolled in current study were reviewed and assessed by other two independent readers in a blind manner which had similar experiences in these fields. For the intra-observer reproducibility, images were reviewed and assessed by the same reader in another extra 30 patients. The interval between the exams was one day.

### Statistical Analysis

The statistical analyses of current study were addressed by SPSS software package (Version 22.0; SPSS Inc, Chicago, IL) and MedCalc software (version 15.2.2, Mariakerke, Belgium). Quantitative data were expressed as mean ± standard deviation (SD) in normal distribution. It was considered to be statistically significant when *p* < 0.05 (two-tailed). Chi-square test or Fisher’s exact test was applied in categorical variables, while independent *t* test was applied in comparison of continuous variables. The malignancy risks for independent variables were calculated in univariate and multivariate analyses. Receivers operating characteristic (ROC) curve analyses were performed to assess the diagnostic performances of conventional US and US elastography. To assess the diagnostic efficacy of combined diagnostic approaches, a series of predicted rates obtained from multivariate logistic regression analysis was subject to ROC curve analysis. The optimal cutoff values for each variable, except BI-RADS, were computed by MedCalc software when Youden Index (YI) was the maximum. Associated areas under the ROC curves (Azs) were compared by *z* test. Intra- and inter-observer reproducibilities were evaluated by Pearson correlation coefficients.

## Results

### Pathologic Diagnosis

In the current study, thirty-four (38.2%) lesions were pathologically confirmed as malignancy, including invasive ductal carcinoma (n = 25), mucinous carcinoma (n = 2), ductal carcinoma *in situ* (DCIS) (n = 3), solid papillary carcinoma (n = 1), intraductal papillary carcinoma (n = 1), malignant phyllodes tumor (n = 1). And the remaining 55 (61.8%) lesions were adenosis (n = 18), fibroadenoma (n = 17), inflammation (n = 10), intraductal papilloma (n = 9), boundary phyllodes tumor (n = 1) and intraductal papilloma with atypical hyperplasia (n = 1). Intraductal papilloma with atypical hyperplasia and boundary phyllodes tumor were regarded as benign lesions in the current study for their less invasiveness and favorable prognosis.

### Univariate analyses on basic characteristics, conventional US and US elastography

**Figure 1:**
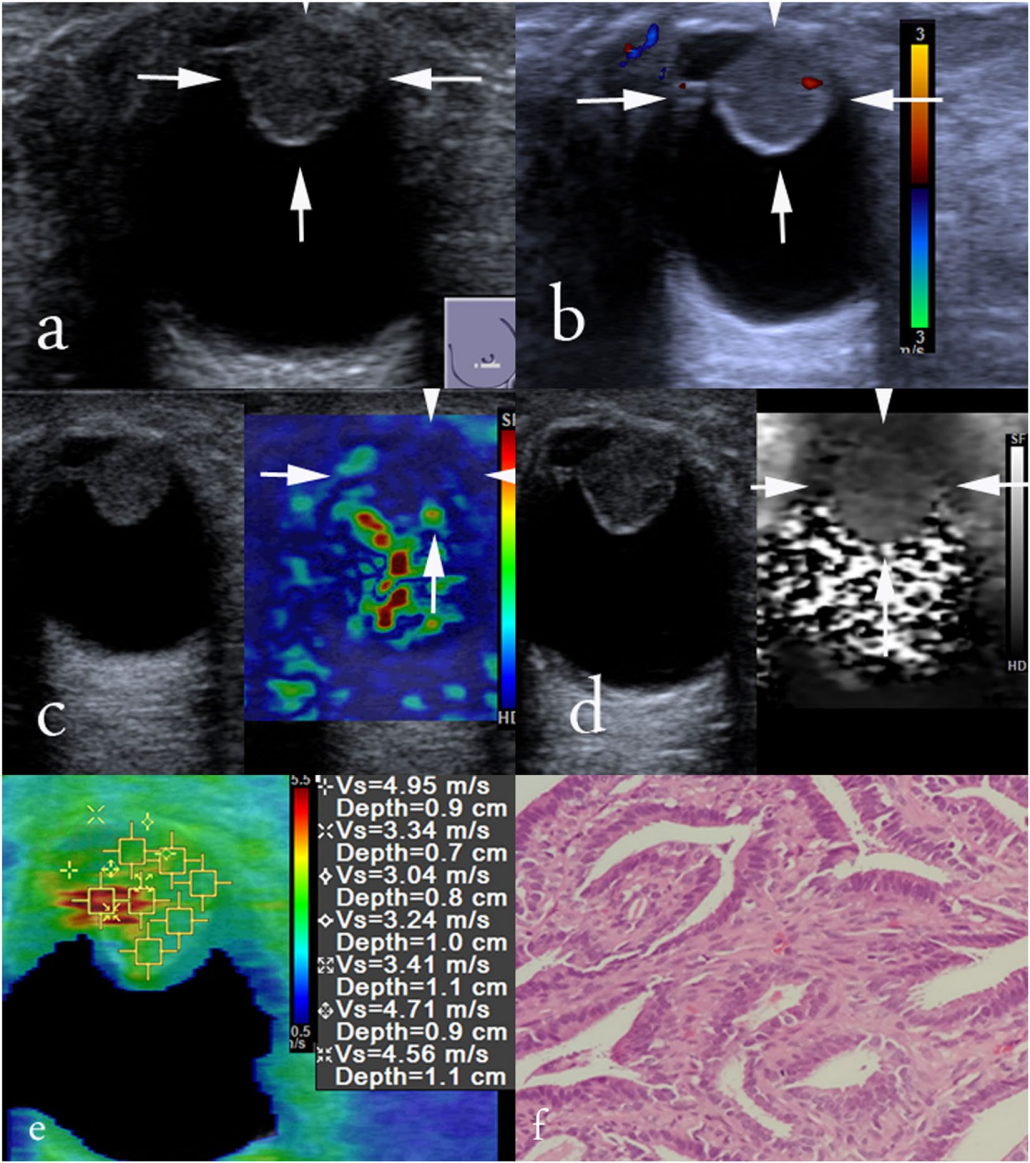
Images in a 45-years-old woman with intraductal papilloma. At conventional US, an 19-mm breast lesion at 6 o’clock position of the left breast appears to be predominately cystic, round in shape, not parallel in orientation, circumscribed in margin, hypoechogenicity, enhancement in posterior acoustic features, absence of internal vascularity and calcification, and was finally classified as BI-RADS category 4a, (**a**) and (**b**). At elastography, SE score of 3 (**c**), pattern 3 in VTI (**d**), and SWS max of 4.95 m/s, SWS median of 3.41 m/s, SWS mean of 3.89 m/s, SWS min of 3.04 m/s (**e**) are obtained. Histologic specimen (hematoxylin-eosin stain; original magnification, × 100) confirms a diagnosis of intraductal papilloma (**f**).

**Figure 2:**
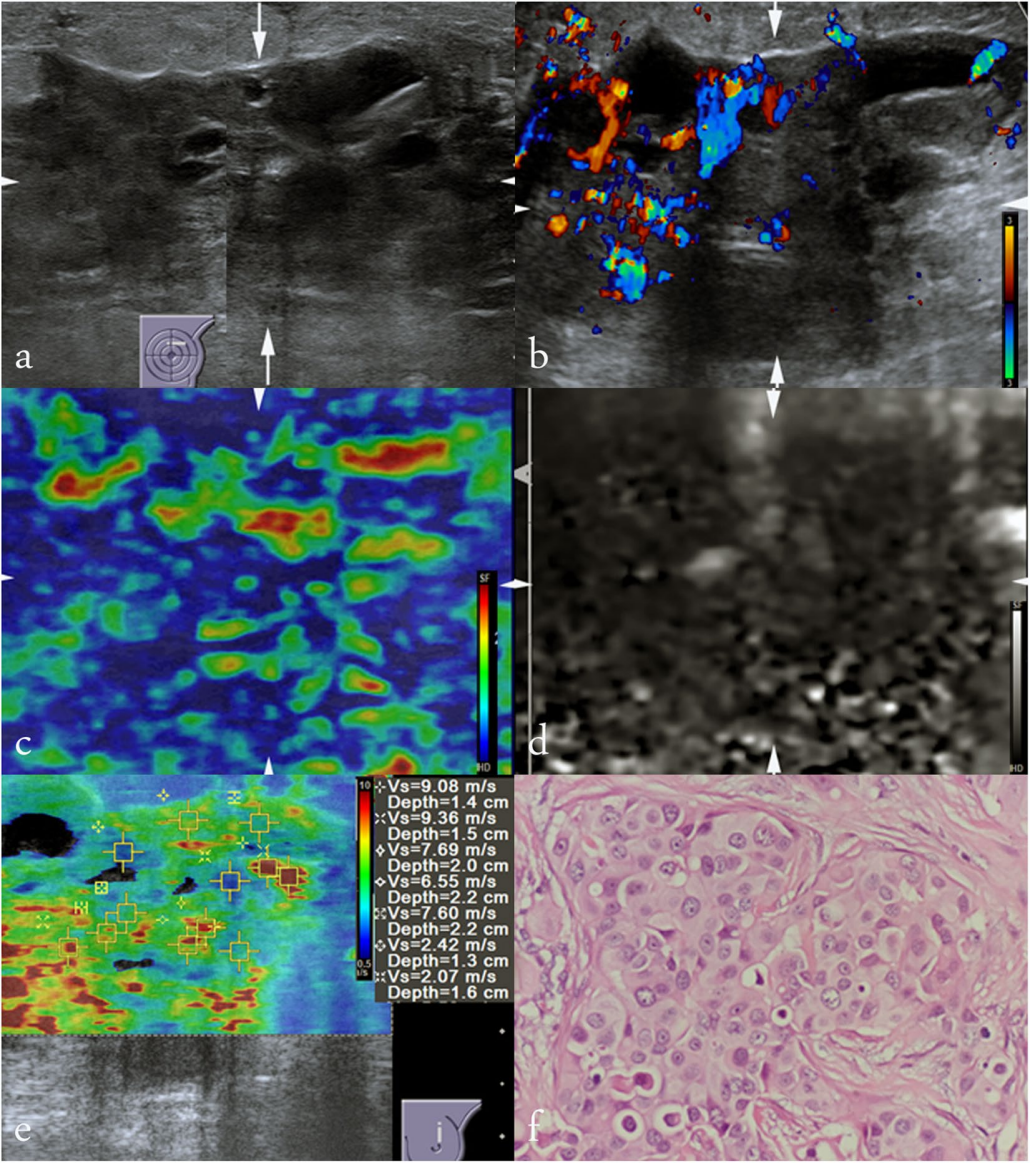
Images in a 42-years-old woman with invasive ductal carcinoma. At conventional US, an 68-mm breast lesion at 12 o’clock position of the left breast appears to be predominately solid, irregular shape, not parallel in orientation, not circumscribed margin, heterogeneous, enhancement in posterior acoustic features, present of internal vascularity, absence of calcification, and is finally classified as BI-RADS category 5, (**a**) and (**b**). At elastography, SE score of 4 (**c**), pattern 4b in VTI (**d**), and SWS max of 9.36 m/s, SWS median of 7.60 m/s, SWS mean of 6.40 m/s, SWS min of 2.07 m/s (**e**) are occurred. Histologic specimen (hematoxylin-eosin stain; original magnification, ×200) confirms a diagnosis of invasive ductal carcinoma (**f**).

As to basic characteristics, the mean patient age was significantly higher in malignancy than that in benign ones (56.3 ± 16.8 yrs vs. 38.7 ± 10.7 yrs; *p* < 0.0001). The optimal cut-off value for patient age obtained from ROC analysis was 50 yrs. Larger lesions were more frequently found in malignancy than in benign lesions (30.1 ± 21.2 mm vs. 21.1 ± 12.3 mm; *p* = 0.012) with the optimal cut-off value of 18 mm. In conventional US characterization, malignant rates were significant higher in groups of cystic component, shape, orientation, margin, echo pattern of solid component, posterior acoustic features and internal vascularity (all *p* < 0.05, detailed *p* value were present in Table 1) (Figs 1 and 2). Internal calcification, however, did not show significant difference between malignant and benign lesions (*p* = 0.057) (Table 1).

**Table 1:** Basic characteristics of the patients and ultrasound features of the lesions.

Characteristics	Benign (n = 55)	Malignant (n = 34)	Malignancy rate (%)	*p* value
**Patient**				
Age (yrs)				<0.0001*
Mean age	38.7 ± 10.7	56.3 ± 16.8	45.4 ± 15.8^a^	
≤50 yrs	45	11	19.6	
>50 yrs	10	23	69.7	
**Lesion**				
Size (mm)				0.012*
Mean size	21.1 ± 12.3	30.1 ± 21.2	24.5 ± 16.7^a^	
≤18 mm	29	9	23.7	
>18 mm	26	25	49.0	
Cystic portion				0.012*
<50%	33	29	46.8	
≥50%	22	5	18.5	
Shape				0.002*
Oval	38	11	22.4	
Round	2	1	33.3	
Irregular	15	22	59.5	
Orientation				0.008*
Parallel	45	19	29.7	
Not parallel	10	15	60.0	
Margin				<0.0001*
Circumscribed	37	3	7.5	
Not circumscribed	18	31	63.3	
Echo pattern of solid portion in masses			<0.0001*	
Hyperechoic	0	0	0.0	
Isoechoic	1	0	0.0	
Hypoechoic	38	10	20.8	
Heterogeneous	16	24	60.0	
Posterior acoustic features^b^		0.035*		
Shadowing	1	5	83.3	
No posterior acoustic features	17	6	26.1	
Enhancement	37	23	38.3	
Internal vascularity^c^				0.006*
Absent	36	12	25.0	
Present	19	22	53.7	
Internal calcification^c^				0.057
Absent	49	25	33.8	
Present	6	9	60.0	

*Indicates statistically significant difference at the level of two-tailed 0.05. ^a^Average value which is obtained from the overall lesions. ^b^No lesion was identified to be combined pattern of posterior acoustic features in current study. ^c^Vascularity and calcification out of a lesion were not referred in current study. n Number.

**Figure 3:**
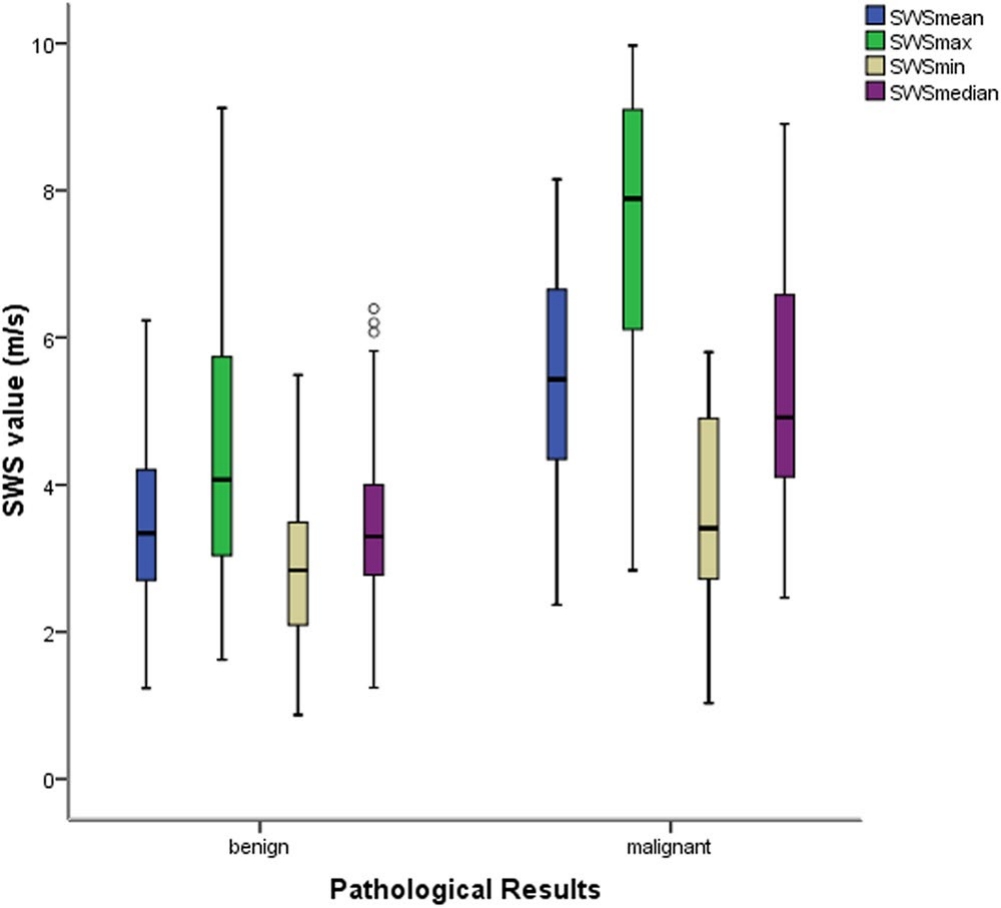
SWS measurements (m/s) are significantly different between benign and malignant lesions (all *p* < 0.05). In benign lesions, SWS max (4.46 ± 1.69, 1.62–9.12), SWS mean (3.54 ± 1.15, 1.23–6.23), SWS median (3.46 ± 1.15, 1.24–6.39) and SWS min (2.83 ± 1.02, 0.87–5.49) are lower. In malignant lesions, SWS max (7.24 ± 2.23, 2.84–9.97), SWS mean (5.34 ± 1.54, 2.37–8.15), SWS median (5.20 ± 1.68, 2.47–8.91) and SWS min (3.62 ± 1.27, 1.03–5.80) are higher.

With respect to the diagnostic approaches in univariate analyses, conventional US, SE, VTI and VTIQ (SWS max-, mean-, median-, min-) were significantly involved in the malignancy prediction (all *p* < 0.05, detailed *p* value were present in Table 2). With the optimal cut-off values, VTI (OR: 21.956; 95% CIs: 5.713–84.383) was revealed to be the most significant variable between malignant and benign lesions, followed by conventional US (OR: 16.054; 95% CIs: 2.031–126.925), SWS max (OR: 14.625; 95% CIs: 5.130–41.692), SWS mean (OR: 14.198; 95% CIs: 4.996–40.346), SE (OR: 12.750; 95% CIs: 3.765–43.173), SWS median (OR: 12.500; 95% CIs: 4.486–34.828), and SWS min (OR: 5.717; 95% CIs: 1.925–16.980) (Table 2, Fig. 3).

**Table 2:** Univariate analyses of conventional ultrasound and ultrasound elastography for malignancy prediction.

Methods	Benign, n	Malignant, n	*p* value
**BI-RADS category**			<0.0001*
3 (n = 19)	18 (94.7%)	1 (5.3%)	
4a (n = 33)	27 (81.8%)	6 (18.2%)	
4b (n = 17)	8 (47.1%)	9 (52.9%)	
4c (n = 9)	1 (11.1%)	8 (88.9%)	
5 (n = 11)	1 (9.1%)	10 (90.9%)	
OR	16.054	95% CI 2.031–126.925	
**SE score**			<0.0001*
1 (n = 5)	5 (100.0%)	0 (0%)	
2 (n = 23)	21 (91.3%)	2 (8.7%)	
3 (n = 23)	15 (65.2%)	8 (34.8%)	
4 (n = 17)	10 (58.8%)	7 (41.1%)	
5 (n = 21)	4 (19.0%)	17 (81.0%)	
OR	12.750	95% CI 3.765–43.173	
**Patterns in VTI**			<0.0001*
1 (n = 6)	6 (100.0%)	0 (0%)	
2 (n = 28)	25 (89.3%)	3 (10.7%)	
3 (n = 25)	16 (64.0%)	9 (36.0%)	
4a (n = 8)	5 (62.5%)	3 (37.5%)	
4b (n = 22)	3 (13.6%)	19 (86.4%)	
OR	21.956	95% CI 5.713–84.383	
SWS max (m/s)	4.5 ± 1.7	7.2 ± 2.2	<0.0001*
>6.0 m/s (n = 34)	8 (23.5%)	26 (76.5%)	
≤6.0 m/s (n = 55)	45 (81.8%)	10 (18.2%)	
OR	14.625	95% CI 5.130–41.692	
SWS median (m/s)	3.5 ± 1.2	5.2 ± 1.7	<0.0001*
>4.2 m/s (n = 34)	9 (26.5%)	25 (73.5%)	
≤4.2 m/s (n = 55)	45 (81.8%)	10 (18.2%)	
OR	12.500	95% CI 4.486–34.828	
SWS mean (m/s)	3.5 ± 1.2	5.3 ± 1.5	<0.0001*
>4.6 m/s (n = 34)	9 (26.5%)	25 (73.5%)	
≤4.6 m/s (n = 55)	46 (83.6%)	9 (16.4%)	
OR	14.198	95% CI 4.996–40.346	
SWS min (m/s)	2.8 ± 1.0	3.6 ± 1.3	0.002*
>3.9 m/s (n = 34)	20 (58.8%)	14 (41.2%)	
≤3.9 m/s (n = 55)	49 (89.1%)	6 (10.9%)	
OR	5.717	95% CI 1.925–16.980	
Total (n = 89)	55 (61.8%)	34 (38.2%)	

*Indicates statistically significant difference at the level of two-tailed 0.05. Numbers in parentheses are percentage of benign and malignant lesions. n, number; BI**-**RADS, Breast Imaging Reporting and Data System; SE, strain elastography; VTI virtual touch tissue imaging; SWS, shear wave speed; OR, Odds Ratios; CI, confidence interval.

### Multivariate Logistic Regression Analysis

In multivariate logistic regression analysis, VTI (OR: 15.278; 95% CIs: 2.495–93.547) was revealed to be the strongest independent predictor for malignancy, followed by lesion margin (OR: 12.346; 95% CIs: 2.242–67.978), SWS mean (OR: 11.896; 95% CIs: 2.619–54.034), and patient age (OR: 6.303; 95% CIs: 1.325–29.974) (Table 3).

**Table 3:** Multivariate logistic regression analysis.

Characteristics	β	SE	OR	95% CI	*p* value
Patient Age	1.841	0.796	6.303	1.325–29.974	0.021*
Margin	2.513	0.870	12.346	2.242–67.978	0.004*
VTI	2.726	0.925	15.278	2.495–93.547	0.003*
SWS mean	2.476	0.772	11.896	2.619–54.034	0.001*

β, regression coefficient; SE, standard error; OR, odds ratio; CIs, Confidence intervals; VTI virtual touch tissue imaging; SWS, shear wave speed. *Indicates statistically significant difference at the level of two-tailed 0.05.

### Diagnostic Performance of conventional US and US elastography

**Figure 4:**
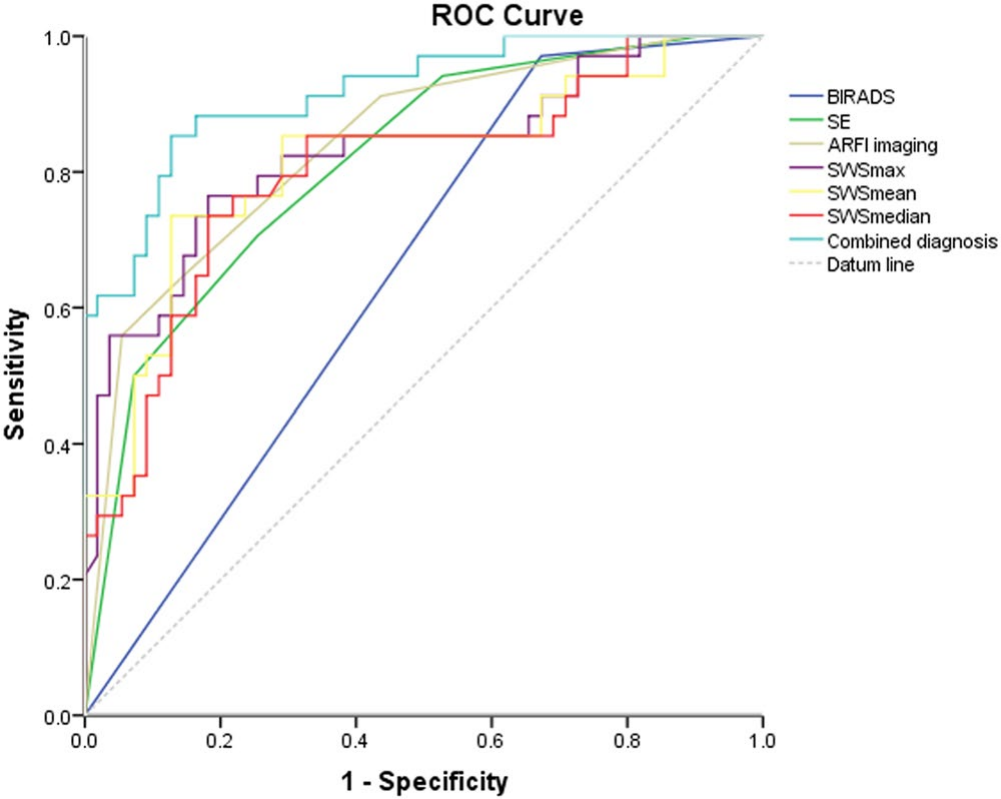
In ROC curve analyses, associated Azs of BI-RADS, SE, VTI, SWS max, SWS mean, SWS median and combined diagnostic method are 0.649, 0.814, 0.840, 0.829, 0.815, 0.801 and 0.918, respectively.

In ROC analyses, the associated Az for BI-RADS category in conventional US was 0.649 (95% CIs: 0.536–0.761) with a sensitivity of 97.1% and a specificity of 32.7%. For US elastography, with optimal cutoff values, VTI (Az: 0.840; 95% CIs: 0.755–0.925) reached the highest diagnostic efficacy, followed by SWS max (Az: 0.829; 95% CIs: 0.735–0.922), SWS mean (Az: 0.815; 95% CIs: 0.717–0.912), SE (Az: 0.814; 95% CIs: 0.724–0.904), SWS median (Az: 0.801; 95% CIs: 0.703–0.900), and SWS min (Az: 0. 677; 95% CIs: 0. 0.561–0.793) (Table 4, Figs 3 and 4).

**Table 4:** The diagnostic performance of US BI-RADS, SE, VTI and VTIQ for malignancy prediction.

Methods	Optimized cut-off value	Sensitivity (%)	Specificity (%)	PPV (%)	NPV (%)	Accuracy (%)	Az (95% CIs)
US BI-RADS	>Category 3	97.1 ^a^ (33/34)	32.7 ^a^ (18/55)	47.1 (33/70)	94.7 (18/19)	57.3 (51/89)	0.649^a^ (0.536–0.761)
SE	Score > 4	50.0 (17/34)	92.7 (51/55)	81.0 (17/21)	75.0 (51/68)	76.4 (68/89)	0.814 (0.724–0.904)
Patterns in VTI	Score > 4	55.9 (19/34)	94.6 (52/55)	86.4 (19/22)	77.6 (52/67)	79.8 (71/89)	0.840 (0.755–0.925)
SWS max	>6.0 m/s	76.5 (26/34)	81.8 (45/55)	72.2 (26/36)	84.9 (45/53)	79.8 (71/89)	0.829 (0.735–0.922)
SWS median	>4.2 m/s	73.5 (25/34)	81.8 (45/55)	71.4 (25/35)	83.3 (45/54)	78.7 (70/89)	0.801 (0.703–0.900)
SWS mean	>4.6 m/s	73.5 (25/34)	87.3 (46/55)	73.5 (25/34)	83.6 (46/55)	79.8 (71/89)	0.815 (0.717–0.912)
SWS min	>3.9 m/s	41.2 (14/34)	89.1 (49/55)	70.0 (14/20)	62.0 (49/79)	70.8 (63/89)	0.677^a^ (0.561–0.793)
Combined diagnostic method	NA	85.3 (29/34)^b^	87.3 (48/55)^c^	80.6 (29/36)	90.6 (48/53)	86.5 (77/89)	0.918^c^ (0.859–0.976)

US, ultrasound; BI-RADS, Breast Imaging Reporting and Data System; SE, strain elastography; VTI virtual touch tissue imaging; VTIQ virtual touch tissue imaging and quantification; SWS shear wave speed, PPV positive predictive value; NPV negative predictive value; Az, Area under the curve; CIs confidence intervals. ^a^ *p* < 0.05 in comparison with SE, VTI, SWS max, SWS median and SWS mean. ^b^ *p* = 0.075 in comparison with BIRADS. ^c^ *p* < 0.05 in comparison with BIRADS.

Conventional US reached the high sensitivity and negative predictive value (NPV) (97.1% and 94.7%, respectively), whereas the specificity and positive predictive value (PPV) were poor (32.7% and 47.1%, respectively). Inversely, SE and VTI achieved the high specificity (92.7% and 94.6%, respectively) while a relatively poor sensitivity (50.0% and 55.9%, respectively) was repaid. VTIQ realized the balance between sensitivity and specificity. SWS values (max-, median-, mean- and min-) were in a sensitivity of 76.5%, 73.5%, 73.5% and 41.2%, respectively, and in a specificity of 81.8%, 81.8%, 87.3% and 89.1%, respectively (Table 4, Figs 3 and 4).

In comparison with other SWS values (max-, median-, and mean-), the diagnostic efficiency in terms of Az of SWS min was significantly lower (all *p* < 0.0001). For that, SWS min was dismissed when combining VTIQ to conventional US in malignancy prediction. As a result of the combined approaches, including conventional US, SE, VTI and VTIQ, the diagnostic efficacy in terms of Az and specificity were significantly elevated, from 0.649 (95% CIs: 0.536–0.761) to 0.918 (95% CIs: 0.859–0.976) (*p* < 0.0001), and from 32.7% to 87.3%, (*p* < 0.0001), without sacrificing sensitivity (97.1% vs. 85.3%, *p* = 0.075) (Table 4, Fig. 4).

### Consistency in conventional US and US elasography among Intra- and Inter-observer

The correlation coefficient value were 0.891, 0.857 and 0.848 for intraobserver and 0.711, 0.701 and 0.691 for interobserver in conventional US, SE and VTI, respectively.

## Discussion

Highly agreeing with the results of Tozaki *et al*., VTI (OR: 15.278; 95% CIs: 2.495–93.547) was revealed to be the most reliable and strongest independent predictor for malignancy in complex cystic and sold breast lesions in the current study^9^. Malignancy risk in lesions identified to be pattern 4b was approximately 15 times as high as that in pattern 1–4a. The result firstly indicated that the induration procedure might start at an early stage of the lesion. Secondly, the surrounding tissue might be early involved in induration procedure of malignancy, even prior to the not circumscribed margin occurred on grey scale image. As an evolution of SE, VTI has overcome the disadvantages in terms of high operator-dependence and low reproducibility, but remain the advantage of entire reflection of tissue stiffness for lesion. That might be the key truth to the current result.

In the present study, not circumscribed margin (OR: 15.952; 95% CIs: 2.242–67.978) was demonstrated to be the second strongest malignancy predictor, following VTI. Not circumscribed margin such as angular, indistinct, microlobulated margin and thick walls have been frequently reported to accompany with malignant complex cystic and solid lesions in various studies^6, 7, 10, 19–24^. It might be ascribed to the invasive behavior resulted from infiltration and aggressivity associated with tumor cells^25^. Not circumscribed margin played a weaker role in malignancy prediction for complex cystic and solid lesions than that in solid lesions (OR: 17.02–31.6)^26, 27^, which might be caused by the morphological tension induced by cystic component.

SWS mean (OR: 11.896; 95% CIs: 2.619–54.034) with a cutoff value of 4.6 m/s was revealed to be the third strongest independent predictor for malignancy. VTIQ might be superior in SWS measurement by taking advantage of smaller ROI and guidance of SW-quality map in comparison with VTQ technique. The contribution made by SWS measurement in malignancy prediction was various in prior studies. For Young modulus, the associated Az of Emax (kpa) for malignancy prediction in complex cystic and solid breast lesions was reported to be 0.968 (95% CI, 0.932–0.985)^28^. A study from Berg *et al*. also indicated that the SWS measurement of Emax has a powerful and promising diagnostic application in solid breast lesions^29^. On the other hand, Li *et al*. advocated a key role that SWSmean played in BI-RADS 4 category lesions^30^. Theoretically, the SWS measurement realized by Young modulus and VTIQ are equivalent. The underlying reason of difference between VTIQ and Young modulus should be ascribed to the various techniques across from various manufactures.

As Booi *et al*. found in cystic breast lesions, the mean patient age in malignancy was substantially higher than that in benign lesions^5^. Moreover, a peak incidence for benign cystic lesions was found in patients with a range of 35–50 yrs^10^. In the current study, with the optimal cutoff value (i.e. 50 yrs), the risk of being malignant in complex cystic and solid breast lesions is around 6 times in older patients (≥50 yrs) as that in younger patients (<50 yrs) (OR: 6.303).

With respect to the diagnostic approaches, conventional US was demonstrated to be an efficient screening method for malignancy prediction in complex cystic and solid breast lesions with a sensitivity of 97.1%. However, its specificity was 32.7%, as low as it was reported previously^31^. On the other hand, though specificities of US elastography were significantly higher than conventional US (all *p* < 0.05), sensitivities were decreased as a sacrifice. Inspiringly, the problem could be overcome by the combination of conventional US and US elastography. When using the combined diagnostic method, the associated Az for malignancy prediction in complex cystic and solid lesion was significantly increased, from 0.649 to 0.918 (*p* < 0.0001), the same as in specificity (from 32.7% to 87.3%, *p* < 0.0001), without sacrificing its sensitivity (97.1% vs. 85.3%, *p* = 0.075). Kim *et al*. has reported that the combination of conventional US and SE could significantly improve the NPV, from 3.8% to 37.1% (*p* < 0.05). To remedy the high operator-dependence and low reproducibility of SE, the current study added qualitative and quantitative elastographical assessment to conventional US in malignancy prediction for complex cystic and solid breast lesions, and gained a greater improvement in diagnostic efficacy as a result.

Several limitations in our study cannot be ignored. First, as a retrospective study, some biases might be mixed up in the case enrollment in current study. Cysts without solid component such as simple cyst, clustered micro-cysts and complicated cysts were excluded for low malignancy risky stratification (0–2%). However, it was reported that only 44% of lesions identified to be complex cystic and solid lesions on conventional US were actually complex cystic and solid lesions^10^. Second, CNB referred in our study seems to be unreliable when being used alone with a documented range of 2–38% for false-negative rate^32^, though the diagnostic efficacy in terms of sensitivity, specificity, PPV (for malignancy, suspicious and atypia) were 88%, 90%, 99% 100% and 80%, respectively^33^. Additionally, 17–27% of lesions in DCIS confirmed by CNB were underestimated^34^. Consequently, it will be more reliable and judgmatic if the long-term follow-up could be complemented into the reference standard in further study. Third, morphological features of lesions may influence readers when they assessing SE or VTI images on the double screen display. Fourthly, the generalizability of our results is highly dependent on the elastography techniques which made it uncertain on the other manufacturers. Finally, the results of current study were concluded from a small number of samples. Large sample size is mandatory in future study.

In summary, the current study demonstrated that pattern 4b in VTI, not circumscribed margin of lesion, SWS mean above 4.6 m/s in VTIQ, and patient age older than 50 yrs were independent malignancy predictors for complex cystic and solid breast lesions. Moreover, combined with US elastography, the diagnostic efficacy of conventional US could be substantially improved.
